# Change in rate of healthcare encounters for respiratory infection from air pollution exposure after improved vehicle emissions standards in New York State

**DOI:** 10.1007/s11869-024-01505-6

**Published:** 2024-01-31

**Authors:** Daniel P. Croft, Mark J. Utell, Han Liu, Shao Lin, Philip K. Hopke, Sally W. Thurston, Yunle Chen, David Q. Rich

**Affiliations:** 1 Department of Medicine, Division of Pulmonary and Critical Care, Strong Memorial Hospital, University of Rochester Medical Center, 601 Elmwood Avenue, Box 692, Rochester, NY 14642-8692, USA; 2 Department of Environmental Medicine, University of Rochester Medical Center, Rochester, NY, USA; 3 Department of Sociology, University at Albany, the State University of New York, Albany, NY, USA; 4 Department of Environmental Health Sciences, University at Albany, the State University of New York, Albany, NY, USA; 5 Department of Public Health Sciences, University of Rochester Medical Center, Rochester, NY, USA; 6 Institute for a Sustainable Environment, and Center for Air Resources Engineering and Science, Clarkson University, Potsdam, NY, USA; 7 Department of Biostatistics and Computational Biology, University of Rochester Medical Center, Rochester, NY, USA

**Keywords:** Air pollution, PM_2.5_, Respiratory infection, Influenza, Sex-based, Adults

## Abstract

The introduction of Tier 3 light-duty vehicles with reduced emissions began in New York State (NYS) in 2017, with required compliance by 2025. We hypothesized that improved air quality during the early implementation of Tier 3 (2017–2019) would result in reduced rates of hospitalizations and emergency department (ED) visits for respiratory infection associated with increased PM_2.5_ compared to 2014–2016 (pre-Tier 3). Using data on adult patients hospitalized or having an ED visit for influenza, upper respiratory infection, culture-negative pneumonia, or respiratory bacterial infection, living within 15 miles of six air quality monitoring sites in NY, and a case-crossover design and conditional logistic regression, we estimated the rates of respiratory infection hospitalizations and ED visits associated with increased ambient PM_2.5_ concentrations in the previous 0–6 days and each week thereafter up to 1 month. Interquartile range (IQR) increases in PM_2.5_ in the previous 6 days were associated with 4.6% (95% CI: −0.5, 10.1) and 11.9% (95% CI = 6.1, 18.0) increased rates of influenza hospitalizations in 2014–2016 and 2017–2019, respectively. This pattern of larger relative rates in 2017–2019 observed at all lag times was only present in males hospitalized for influenza but not other infections or in females. The rates of respiratory infection visits associated with increased PM_2.5_ were generally not reduced in this early Tier 3 implementation period compared to 2014–2016. Limited fleet penetration of Tier 3 vehicles and differences in particle deposition, infection type, and sex by period may all have contributed to this lack of improvement.

## Introduction

Influenza and bacterial pneumonia together are the ninth leading cause of death in the USA (Santo et al. 2021), and ambient air pollution has been identified as a risk factor for both respiratory viral and bacterial infections worldwide (Kirwa et al. 2021). While pandemic viruses such as SARS-CoV-2 have recently dominated current international attention, seasonal respiratory viral infections (RVIs), ranging from severe influenza to less severe upper respiratory tract infections (URIs), represent an ongoing threat to health. Individuals with RVI may require hospitalization and can even suffer from bacterial superinfection leading to bacterial pneumonia (Rynda-Apple et al. 2015). Thus, understanding the risk factors for hospitalization from acute upper and lower respiratory tract infections is a crucial step in reducing the worldwide burden of disease from both respiratory viral and bacterial infections.

We and others have reported increased rates of respiratory infections associated with short-term increases in ambient air pollution (Croft et al. 2019; Horne et al. 2018; Renzi et al. 2022; Zhang et al. 2023). Previously in New York State, the rate of respiratory infection hospitalizations/emergency department (ED) visits associated with interquartile range (IQR) increases in PM_2.5_ concentration were generally largest in the 6 or 7 days before the hospitalization/ED visit (e.g., influenza: excess rate = 3.9%; 95% CI: 2.1, 5.6) compared to earlier time periods (1 day prior to influenza diagnosis: excess rate = 0.5%; 95% CI: −0.8, 1.8) (Croft et al. 2019). In contrast to a 1 week lag window, Horne et al. examined pollutant concentrations up to 4 weeks before the hospitalization/ED visit, and often reported the largest effect estimates for PM_2.5_ concentrations 4 weeks before hospitalization (e.g., acute lower respiratory infection: odds ratio = 1.2; 95% CI: 1.1, 1.3) (Horne et al. 2018). More recently, the Multiethnic Study of Atherosclerosis (MESA) study in six US regions reported a 4% higher risk of outpatient respiratory infection (95% CI = 0, 9) associated with each 5.7 μg/m^3^ increase in PM_2.5_ concentration in the preceding 2–6 weeks (composite lag time) and a 21% increase in the risk of respiratory infection (95% CI = 10, 33) associated with each 14.4 ppb increase in NO_2_ concentration in the preceding 2–6 weeks (Kirwa et al. 2021). A study in China, with laboratory-confirmed influenza infection, reported a 3.8% increased risk of influenza (95% CI = 1.6, 6.0) associated with each 50 μg/m^3^ increase in PM_2.5_ in the previous 5 days (Lu et al. 2020). Although our prior work focused only on lower respiratory infections (i.e., influenza and pneumonia), other studies reported associations between increased concentrations of ambient air pollutants and upper respiratory tract infections (Chen et al. 2018; Horne et al. 2018).

Over the past decade, the state of New York has undergone changes in air quality as a result of upwind coal-fired power plant closures, changes from coal to natural gas for energy generation, reduction in sulfur concentrations in distillate fuels, the North American Emissions Control Area (NA-ECA), and the phase-out of residual oil for space heating in New York City (Chen et al. 2023). In addition, economic drivers such as the 2007–2009 recession and the change in the relative costs of natural gas and coal drove changes in the mixture of electricity-generating unit technologies and downwind air quality. While these changes resulted in reduced PM concentrations across New York State, a change in PM composition also occurred (i.e., the proportion of PM from secondary organic carbon and spark-ignition vehicle pollution increased from 2006 to 2016 (Masiol et al. 2019)). Thus, despite this reduction in PM_2.5_ concentration, the risk for hospitalization and ED visits for respiratory infection (e.g., influenza ED visits) associated with each 5.7 μg/m^3^ increase in PM_2.5_ concentration was higher in 2014–2016 (excess rate = 6.4%, 95% CI: 2.8, 10.1) than in 2009–2013 (excess rate = 2.1%, 95% CI 4.2, 20.1). This suggested that the toxicity of the PM per unit mass may have increased in New York from 2005–2016. In 2017, new regulations improving emissions of Tier 3 light-duty vehicles began in NY State, with all vehicles mandated to meet this standard by 2025 (Hopke and Hidy 2022). The expected reduction in tailpipe and evaporative emissions from the Tier 3 program provided an opportunity to determine if the rate of hospitalization or ED visits for respiratory infection associated with increased PM concentrations was reduced after these Tier 3 vehicle regulations were implemented in 2017.

Using a New York statewide database of respiratory infection healthcare encounters paired with ambient air pollution concentrations at monitoring stations in Buffalo, Rochester, Albany, Manhattan, Queens, and the Bronx from 2014 to 2019, we estimated the rate of healthcare encounters for upper and lower respiratory infection associated with increased air pollution concentrations in the previous few days and weeks for adult residents of New York State. Our study examined the association between PM_2.5_ and respiratory infection healthcare visits and whether these relative rates were reduced during the early implementation of the Tier 3 emissions controls. Specifically, we compared the 2014–2016 period to the 2017–2019 period, hypothesizing that the introduction of Tier 3 vehicles into the light-duty fleet would result in a lower rate of hospitalization and ED visits from respiratory infection associated with increased PM in 2017–2019 than 2014–2016.

## Methods

### Study population

Respiratory infection hospital admissions and ED visits for adult New York residents were obtained from the Statewide Planning and Research Cooperative System (SPARCS) database. In total, *N* = 135,236 hospitalizations and *N* = 549,528 ED visits of adults living within 15 miles of the Buffalo, Rochester, Albany, Bronx, Manhattan, or Queens PM_2.5_ monitoring sites from January 1, 2014. to December 31, 2019. were retained. The 15-mile radius was selected to be consistent with our prior study in New York State (Croft et al. 2019). This distance balances the tradeoff between minimizing exposure misclassification and optimizing sample size (Zikova et al. 2017). We included participants with a primary diagnosis (at time of healthcare encounter) of influenza (ICD9 = 4870, 4871, 48811, 48812, 48881, 48882; ICD10 = J09X1, J09X2, J1000, J1001, J1008, J101, J1100, J1108 and J111, bacterial pneumonia (ICD9 = 481, 482, 4830, 4831; ICD10 = J13, J14, J15, J16, A48.1) or culture-negative pneumonia (ICD9 = 485, 486; ICD10 = J18). Culture-negative pneumonia is a common diagnosis, as modern culture techniques identify a causative pathogen in < 50% of the patients diagnosed with pneumonia (Purcaro et al. 2018). It is best viewed as an undifferentiated infection that can be bacterial or viral in origin. This study was approved by the Institutional Review Board at the University at Albany, State University of New York.

### Air pollution and weather

From 2014 to 2019, hourly PM_2.5_ concentrations at the Buffalo, Rochester, Albany, Bronx, Manhattan, and Queens monitoring stations were retrieved from the US Environmental Protection Agency (https://aqs.epa.gov/api). Additional details on the monitor types used for PM_2.5_ measurements and the measurements of temperature and relative humidity have been described previously (Chen et al. 2023). Each participant was assigned daily PM_2.5_, temperature, and relative humidity values from the monitoring station closest to their residence.

### Statistical analysis

We used a time-stratified, case-crossover design (Maclure 1991; Levy et al. 2001) to estimate the rates of respiratory infection hospital admissions and emergency department (ED) visits associated with each interquartile range increase in PM_2.5_ concentration on the same day (lag day 0). For influenza hospital admissions from all six urban sites, assuming a common slope across sites, we fit a conditional logistic regression model stratified on each respiratory infection hospital admission matched set (1 case and 3–4 control periods per subject). This conditional logistic regression model regressed case-control status (i.e., case = 1, control = 0) against the mean PM_2.5_ concentration on case and control days. The case-crossover approach controls for non-time-varying confounders, such as underlying medical conditions, patient insurance status, long-term time trends, and season, by design. However, we included natural splines for temperature and relative humidity (4 degrees of freedom determined using the Akaike information criterion) (Aho et al. 2014). This same model was run for the mean PM_2.5_ concentration on lag days 0–1, 0–2, 0–3, 0–4, 0–5, 0–6, 7–13, 14–20, and 21–27, and then separately for ED visits and hospitalizations for URI, bacterial pneumonia, and culture-negative pneumonia. From each model, we estimated the rate of hospitalizations or ED visits associated with each interquartile range increase in PM_2.5_ concentration. Since we examined ten lag times for each disease subgroup, statistical significance was defined as *p* < 0.005 (0.05/10).

Next, we examined whether the rates of respiratory infection admission associated with each interquartile range increase in PM_2.5_ concentration differed by period (2014–2016 and 2017–2019) by adding an interaction term (period * PM_2.5_) to the model. Last, we stratified by reported sex (males versus females) and re-ran the above models separately for adult males and females. All analyses were done using R version 3.0.1 (https://www.r-project.org/).

## Results

The majority of participants requiring hospitalization or ED visits were female (54% and 62%, respectively), with hospitalized patients generally being older (mean age = 68 years old) than patients treated in the ED only (mean age 40 years old) (Table 1). Of the participants hospitalized, 48% were white and 25% were black, while 27% and 39% of participants treated in the ED were white and black, respectively. The majority of hospitalized participants had culture-negative pneumonia (66%), while the majority of participants treated and released from the ED suffered from upper respiratory tract infections (79%). Hospitalized participants stayed in the hospital for approximately 5 days on average. Predictably, more infections occurred in the winter and spring (60%) in both care settings than in the summer and fall (40%). Among control days, Manhattan had the highest median PM_2.5_ concentration (8.2 μg/m^3^), while Albany had the highest maximum PM_2.5_ concentration (45.4 μg/m^3^) (Table 2). Rochester had both the lowest median and lowest maximum PM_2.5_ concentration (5.9 μg/m^3^ and 24.8 μg/m^3^ respectively). There were similar patterns among case periods.

**Fig. 1:**
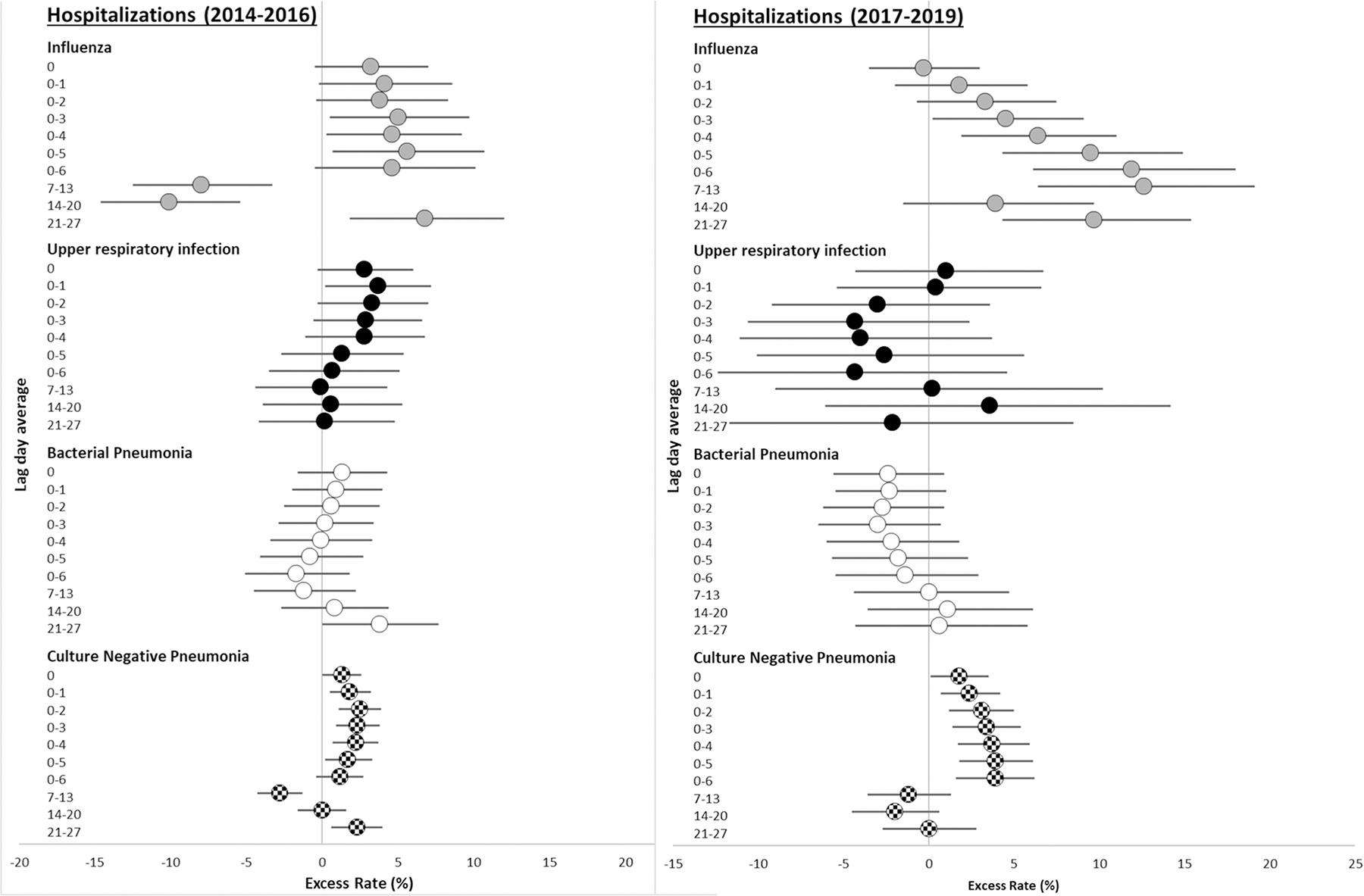
Excess rate of hospitalization for influenza, upper respiratory infection, bacterial pneumonia, and culture-negative pneumonia associated with each IQR increase in PM_2.5_ in 2014–2016 and 2017–2019

**Fig. 2:**
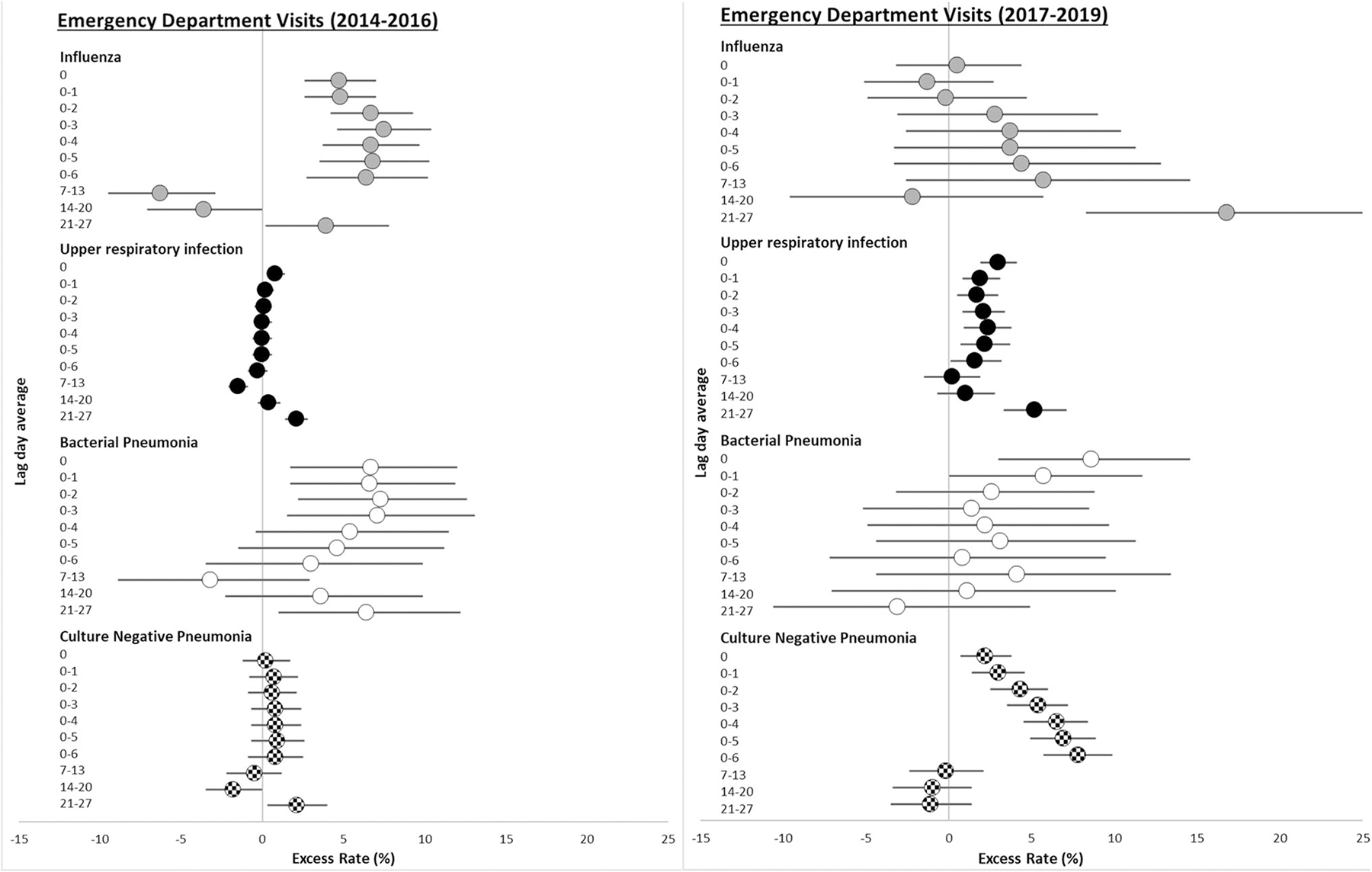
Excess rate of ED visits for influenza, upper respiratory infection, bacterial pneumonia, and culture-negative pneumonia associated with each IQR increase in PM_2.5_ concentration in 2014–2016 and 2017–2019

Rates of influenza hospitalizations and ED visits associated with interquartile range (IQR) increases in PM_2.5_ concentrations were largest in the previous 4, 5, and 6 days prior to presentation, throughout the entire study period 2014–2019 (Table 3). Although not significantly different, influenza hospitalization rate ratios were generally larger in the 2017–2019 period compared to the 2014–2016 period (Fig. 1). For example, each 4.1 μg/m^3^ increase in PM_2.5_ in the previous 6 days was associated with an 11.9% increased rate of influenza hospitalization (95% CI = 6.1, 18.0) in the 2017–2019 period, but only a 4.6% increased rate (95% CI: −0.5, 10.1) during the 2014–2016 period. However, in the 2014–2016 period, each interquartile range increase in PM_2.5_ concentration in the previous 7–13 days was associated with an 8.0% decrease in the rate of influenza hospitalizations (95% CI = −12.5, −3.3) and a 6.3% decrease in the rate of influenza ED visits (95% CI = −9.5, −2.9). In contrast, in 2017–2019, each interquartile range increase in PM_2.5_ was associated with a 12.6% increase in the rate of influenza hospitalization (95% CI = 6.4, 19.1) (Fig. 1) and a 5.7% increase in the rate of influenza ED visits (95% CI = −2.6, 14.6) (Fig. 2).

**Fig. 3:**
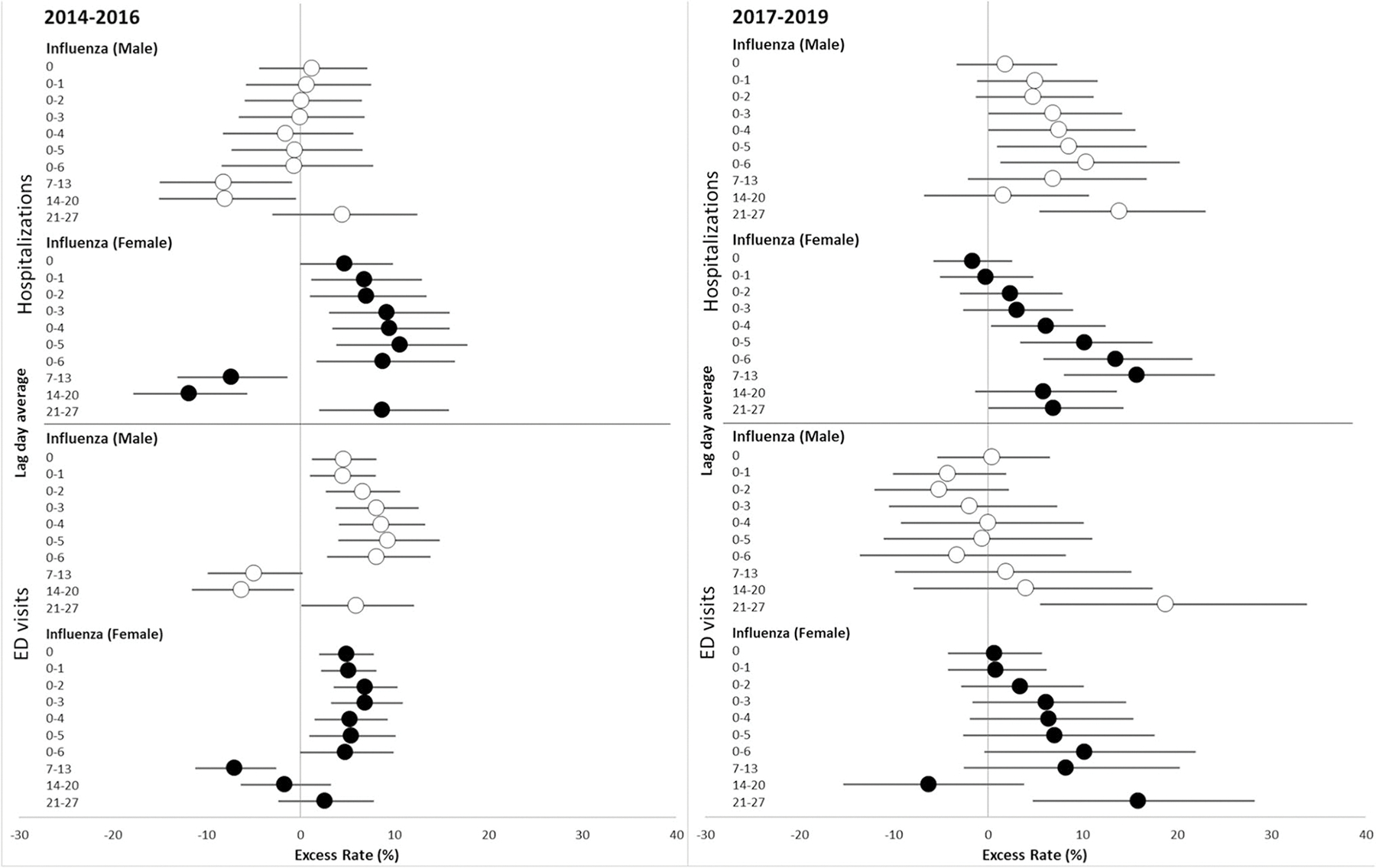
Excess rate of hospitalization and ED visits for influenza, associated with each IQR increase in PM_2.5_ concentration in 2014–2016 and 2017–2019 by sex

There were not substantial differences in influenza admissions and ED visit excess rates between males and females. Both males and females generally had the highest excess rates of influenza hospitalization associated with interquartile range increases in PM_2.5_ concentration in the previous 4, 5, and 6 days in both the 2014–2016 and 2017–2019 periods (Table 4)(Fig. 3). However, for males, the 2017–2019 influenza excess rates were substantially larger than in the 2014–2016 period. For females, excess rates were not consistently different between the 2014–2016 and 2017–2019 periods. Specifically, for males in 2014–2016, each IQR increase in PM_2.5_ concentration in the previous 5 days was associated with a 0.6% decrease in the rate of hospitalization for influenza (95% CI = −7.4, 6.6) compared to an 8.6% increase in the rate of hospitalization (95% CI = 0.9, 16.8) in the 2017–2019 period. For females, excess rates of hospitalization associated with interquartile range increases in PM_2.5_ concentration for the 2014–2016 and 2017–2019 periods were similar (lag days 0–4: 10.6 and 10.2, respectively). For influenza ED visits in males, the excess rates appeared smaller in the 2017–2019 period than in 2014–2016, while the excess rates for females between periods were not consistently different.

Rates of upper respiratory infection ED visits associated with IQR increases in PM_2.5_ concentrations in the previous few days and weeks were larger in the 2017–2019 period than in the 2014–2016 period (Table 3). For example, each IQR increase in PM_2.5_ concentration in the previous 5 days was associated with a 2.4% increase in the rate of ED visits for upper respiratory infection (95% CI = 0.9, 3.8) in the 2017–2019 period, but not the rate of upper respiratory infection ED visits (excess rate = 0.0%; 95% CI: −0.6, 0.6) in the 2014–2016 period. Upper respiratory infection hospitalization excess rates were not different between the 2017–2019 period and the 2014–2016 period, and there were no differences in upper respiratory infection excess rates between males and females (Table S1).

There were no associations between bacterial pneumonia hospitalizations and increased PM_2.5_ concentrations in the previous few days and weeks, in either the 2014–2016 period or 2017–2019 period, with excess rates ranging from −3.0 to 3.8% (Table 3 and Fig. 1). However, there were similarly sized to slightly decreased excess rates of bacterial pneumonia ED visits associated with IQR increases in PM_2.5_ concentrations in the 2017–2019 period compared to the 2014–2016 period. For example, each 4.0 μg/m^3^ increase in PM_2.5_ concentration in the previous day was associated with a 7.3% increase in the rate of bacterial pneumonia ED visits (95% CI: 2.2, 12.6) in the 2014–2016 period, with a smaller 2.6% excess rate in the 2017–2019 period (95% CI: −3.2, 8.8). In separate analyses for males and females, there were generally no associations between the rate of bacterial pneumonia associated with IQR increases in PM_2.5_ concentration in the previous few days and weeks in either the 2014–2016 or 2017–2019 period. There were also no consistent differences between period-specific excess rates for males and females (Table S2).

Interquartile range increases in PM_2.5_ concentration in the previous 1–7 days were generally associated with increased rates of culture-negative pneumonia hospitalizations in both the 2014–2016 and 2017–2019 periods, although the excess rates were slightly larger in the 2017–2019 period (Fig. 1, Table 3). For example, each 4.2 μg/m^3^ increase in PM_2.5_ concentration in the previous 3 days was associated with a 2.5% (95% CI: 1.1, 3.9) increased rate of culture-negative pneumonia hospitalization in the 2014–2016 period, and a 3.1% increased rate (95% CI: 1.2, 5.0) in the 2017–2019 period. For culture-negative pneumonia ED visits, excess rate estimates were generally larger in the 2017–2019 period (Fig. 2). For example, each 3.2 μg/m^3^ increase in PM_2.5_ concentration in the previous 7 days was associated with a 7.8% excess rate in the 2017–2019 period (95% CI = 5.7, 9.9), but not the 2014–2016 period (excess rate = 0.8%; 95% CI: −3.5, 0.0). There were no sex differences in excess rates of culture-negative pneumonia hospitalizations and ED visits across periods (Table S3).

## Discussion

As expected, increased ambient PM_2.5_ concentrations in the previous 7 days were associated with increased hospitalizations and ED visits for influenza and culture-negative pneumonia and ED visits for upper respiratory infection among adults living in Buffalo, Rochester, Albany, and New York City, New York, from 2014 to 2019. These associations were independent of concurrent changes in temperature and relative humidity, as well as any subject-specific demographic and clinical characteristics that did not vary over the study period. Inconsistent with our *a priori* hypothesis, rates of influenza hospitalizations, upper respiratory infection ED visits, and culture-negative pneumonia hospitalizations and ED visits associated with increased PM_2.5_ concentrations were larger in the 2017–2019 period (i.e., after the early implementation period of Tier 3 vehicle introduction) than in the 2014–2016 period. However, for other infection hospitalization and ED visit categories, we did not see this pattern. Finally, outside of differences in influenza infections, there were no consistent differences in cause-specific excess rates by sex.

Our finding of increased rates of respiratory infection (i.e., influenza, upper respiratory infection, bacterial pneumonia, and culture-negative pneumonia) hospitalization and ED visits associated with increased ambient PM_2.5_ concentrations in the previous few days and weeks is consistent with our and others previous work (Croft et al. 2019; Horne et al. 2018; Pirozzi et al. 2018; Kirwa et al. 2021; Zhang et al. 2023). However, our study also compared the size of any PM_2.5_/hospitalization-ED visit associations between the 2014–2016 period and the 2017–2019 period. Despite air pollution concentration reductions from an average PM_2.5_ concentration of 7.9 μg/m^3^ in the 2014–2016 period to an average PM_2.5_ concentration of 7.0 μg/m^3^ in the 2017–2019 period (Chen et al. 2023), the excess rate of healthcare contacts for respiratory infection associated with PM_2.5_ concentrations in our study did not decrease. In our prior study among New York State adults from 2005 to 2016, we found that each 5.7 μg/m^3^ increase in PM_2.5_ concentration in the previous 6 days was associated with a 6.4% increase in influenza ED visits in the 2014–2016 period, but only a 2.1% increase from 2008 to 2013. Since we observed a decrease in PM_2.5_ and other pollutant concentrations from 2005 to 2016 (Squizzato et al. 2018), we hypothesized that an increase in PM toxicity per unit mass concentration (i.e., the change in the chemical composition of PM may have made the same mass concentration more toxic) may have occurred following the series of air quality regulations and economic drivers occurring from 2008 to 2013 (Croft et al. 2019). A reason for the increased toxicity would be an increase in oxidative potential (reactivity) of PM_2.5_. An increase in oxidative potential may contribute to a decrease in macrophage-mediated phagocytosis and clearance of pathogens, possibly increasing the severity of infection (Wu et al. 2018). Similar to this prior study (Croft et al. 2019), our current findings when comparing respiratory infection relative rates associated with PM_2.5_ between 2014–2016 and 2017–2019 are not consistent with our a priori hypothesis and suggest that the current market penetration of the Tier 3 vehicle introductions in 2017 may not have a measurable respiratory infection health benefit in New York State. Specifically, despite the decreases in PM concentrations from 2014 to 2019 (Chen et al. 2023), we did not observe a decrease in the rate of healthcare encounters for respiratory infection associated with PM. While the exact percentage of market penetration of Tier 3 vehicles into the New York fleet is not yet known, a repeated assessment of this association will be helpful in understanding the full impact of the Tier 3 policy.

In addition to our prior concern of increased toxicity per unit mass of PM_2.5,_ there are several other possible explanations for the trends we observed in the excess rate of respiratory infection. First, the increased excess rate of influenza hospitalizations (but no change in influenza ED visit relative rates) in the 2017–2019 period compared to the 2014–2016 period may indicate that the severity of health care encounters for influenza associated with PM increased between periods. Nationally, there were 22 million (19%) more symptomatic influenza illnesses in the USA, 260,000 (15%) more influenza hospitalizations, and also 7000 (5%) fewer influenza deaths than in the 2014–2016 period (CDC 2023). While there were more illnesses in the 2017–2019 period, fewer deaths would support a potentially more infectious but less lethal strain of influenza being present. Overall, while more influenza infections occurred in 2017–2019 than in 2014–2016, this is unlikely to fully explain the observed increased rate of influenza hospitalization and ED visits associated with increased ambient PM_2.5_ concentrations in the previous few days among New York adults during this time period.

The proportion of particles smaller than 500 nm comprised of ultrafine particles (UFP) increased from the 2014–2016 period to the 2017–2019 period, particularly in the 10–20 nm and 20–50 nm particle sizes (Chen et al. 2023). The increase in smaller particle formation (< 50 nm) may be due to reductions in the concentration of accumulation mode particles (AMP; 100–500 nm), which serve as a condensation sink for condensable substances such as UFP (Chen et al. 2023). It appears unlikely that the change in Tier 3 standards or even prior gasoline direct injection (GDI) would result in a decrease in AMP concentration, although prior GDI standard changes to meet fuel efficiency standards may have contributed to an increase in small particle generation (Yi et al. 2022). The smaller UFP particles (< 50 nm) are known to predominately deposit by diffusion (Brownian motion) in the upper airways (upper trachea/posterior pharynx). However, larger UFP particles (≥ 50 nm) have a weaker deposition (diffusional or inertial) in the upper or lower airways (Oberdörster et al. 2005). The deposition of the smaller UFP particles (< 50 nm) in the upper respiratory tract may lead to a greater risk of influenza and upper respiratory infection associated with increased PM_2.5_, compared to lower respiratory tract bacterial infection, given the disruption of the mucosa in the upper airway.

Influenza is a particularly dangerous infection, as it can spread from the initial site of infection in the upper airway down to the lower airway to cause pneumonia (whether influenza alone or a bacterial superinfection of influenza). While upper respiratory infections also have an initial site of infection in the upper airway, upper respiratory infections typically lead to less severe infections due to the lower likelihood of spreading to the lower airways. As expected, we observed null associations between PM and upper respiratory infection hospitalizations in both periods. However, we observed larger ED visit excess rates for upper respiratory infections in the 2017–2019 period than in the 2014–1016 period. This pattern may also be related to the increased UFP component of PM_2.5_, increasing the susceptibility to URIs.

The lack of clear differences in the association between bacterial pneumonia and PM_2.5_ between periods may reflect the change in deposition location (i.e., being in the upper airway rather than in the lower airway where bacterial pneumonia typically originates). The increased magnitude of excess rates for culture-negative pneumonia ED visits in the 2017–2019 period compared to 2014–2016, and no clear change in hospitalization excess rates was different from all other types of infections. This may be related to the fact that culture-negative pneumonia are comprised of both undiagnosed bacterial and viral infections that are likely unique from influenza or typical bacterial pneumonia organisms. While the type of infection is likely an important factor in whether an association between PM and respiratory infection is observed, the PM deposition location (related to its composition) may be another important factor to consider.

While distinct sex-specific immune responses to respiratory viral infection have been observed previously (Ghosh and Klein 2017; Silveyra et al. 2021), a recent study focused on the sex-specific response to respiratory infection in the setting of a controlled wood smoke exposure (Rebuli et al. 2019). Specifically, in this randomized controlled trial of a 2-h chamber exposure to 500 μg/m^3^ of wood smoke particles followed by exposure to a live attenuated influenza virus, the authors observed a greater nasal mucosal inflammatory gene response in adult males compared to females. In our study, females had the highest excess rates of influenza and bacterial pneumonia in both periods, though males had the larger increase in influenza hospitalization excess rates between the two periods. One possible explanation for this could be that the composition of PM in 2017–2019 led to a greater inflammatory effect in males with influenza, compared to the exposure in 2014–2016, while not changing the risk of respiratory viral infection admission in females. In addition to the need to study sex-specific effects, determining the effect of air pollution on specific types of infections rather than general groups is another important focus area for future research. Secondly, we did not have access to viral or bacterial respiratory infection diagnoses in outpatient clinics and physician offices. This may have led to an underestimation of the counts of healthcare encounters with a comparable severity to ED visits for upper respiratory infections and to a lesser degree, influenza ED visits during each of the respective time periods.

Although this study had several important strengths, including large sample size and its resulting enhanced statistical power, use of the case-crossover study and its control of non-time varying subject characteristics/confounders and any interactions between them by design, and a statewide comprehensive dataset of health care encounters for respiratory infection, there are several weaknesses that should be considered when making inference. First, study subjects were assigned PM_2.5_ concentrations from the monitoring station closest to their home, no matter if they lived near the monitor or as far as 15 miles from the station (or spent most of the day at work in a different community). However, this non-differential exposure misclassification should just result in the underestimation of excess rates. Second, in our period analyses, we were not able to control for the potential differences in influenza or *Streptococcus pneumoniae* vaccine status of participants, influenza strain variation, or the number of illnesses from influenza between the two periods.

Last, the use of the case-crossover design and its time-referent selection strategy (i.e. control periods matched to case periods by weekday within the same calendar month and year) (Levy et al. 2001)) allows estimation of the rate of infection associated with increased PM_2.5_ concentrations in the previous 1 to 7 days, keeping case and control period dates and PM_2.5_ concentrations separate (i.e., the dates of the case period are completely separate from the dates of the control periods for each participant). However, when this design is applied to estimate the rate of infection associated with increased PM_2.5_ concentrations in the previous weeks (e.g., lag days 7–13, 14–21), as we did in this study and others have done previously (Horne et al. 2018), calendar dates that were included as “case periods” in one lagged analysis (e.g., lag days 0–6, 0–5, 0–4) may now be included as “control periods” in a second lagged analysis (e.g., lag days 7–13, 14–21). For example, assume we are interested in estimating the rate of influenza hospitalization associated with increased PM_2.5_ concentrations in the previous 7 days (lag days 0–6). If the influenza hospitalization for a hypothetical subject occurred on Thursday, November 15, 2018, we would contrast the PM_2.5_ from the case period (November 9–15) with PM_2.5_ concentrations from 4 control periods (November 23–29, November 16–22, November 2–8, and October 25–November 1). Now, as part of the same study, assume we also want to estimate the rate of influenza hospitalization associated with increased PM_2.5_ concentrations in the prior week (e.g., lag days 7–13). For that same subject influenza hospitalization, we would contrast the PM_2.5_ from the case period (November 2–8) with PM_2.5_ concentrations from 4 control periods (November 16–22, November 9–15, October 25–November 1, and October 19–25). However, what was a case period in the lag day 0–6 analysis (November 9–15) is now a control period in the lag day 7–13 analysis, and what was a control period in the lag days 0–6 analysis (November 2–8) is now a case period in the lag days 7–13 analysis. Thus, if there is any association between PM_2.5_ and the rate of influenza hospitalization, effect estimates for these two different lagged analyses would be expected to be quite different. If one effect estimate is > 1.0, you may expect the other effect estimate to be < 1.0, and thus, making inferences on the triggering of influenza by short-term increases in PM_2.5_ in these later weeks may be problematic. In our analyses in the 2014–2016 period, we found that each IQR increase in PM_2.5_ concentration in lag days 0–6 was associated with a 4.6% increase in the rate of influenza hospitalizations, but an IQR increase in PM_2.5_ concentration in lag days 7–13 was associated with an 8.0% decrease in the rate of influenza hospitalization. The large difference in effect size between these two lagged influenza hospitalization analyses (positive association to negative association) may, in part, be a result of this referent selection problem for lag times longer than 7 days. Thus, we suggest future analyses use a modified case-crossover referent selection algorithm without such sharing of dates and PM_2.5_ concentrations or preferably use a different study design to examine lag times longer than 7 days.

## Conclusions

Despite the introduction of Tier 3 vehicles with lower pollutant emissions in 2017, the excess rates of influenza, URI, bacterial pneumonia, and culture-negative pneumonia hospitalizations and ED visits associated with increased PM_2.5_ concentrations in the previous 7 days in 2017–2019 were not reduced in this early Tier 3 implementation period, compared to 2014–2016. It is possible that an increase in the proportion of the ultrafine component of PM_2.5_ may have led to an increase in toxicity per unit mass of PM_2.5_. Due to deposition in the upper airway, it is possible that increases in ultrafine particles contributed to an increased rate of influenza hospitalizations but did not contribute to the rate of bacterial pneumonia hospitalizations. The increase in the influenza hospitalization excess rate associated with increased PM_2.5_ in the 2017–2019 period compared to the 2014–2016 period appeared to be primarily driven by an increase in hospitalization in males. In summary, limited penetration of Tier 3 vehicles to the New York State vehicle fleet, pollution deposition location in the lung, type of infection, and differences in relative rates by sex may all have contributed to the lack of improvement in respiratory infection rates associated with increased PM after Tier 3 emissions regulations were implemented.

## Supplementary Material

Supplement

**Supplementary information** The online version contains supplementary material available at https://doi.org/10.1007/s11869-024-01505-6.

## Figures and Tables

**Table 1:** Characteristics of respiratory infectious disease hospital admissions and ED visits (2014—2019), by study site/city

	Hospitalizations (*N* = 135,236)		ED visits (*N* = 549,528)	
Characteristic	*n*	%	*n*	%

INFECTION TYPE				
Influenza	15,837	12	28,647	5
Upper respiratory tract infection	12,100	9	436,411	79
Bacterial pneumonia	17,505	13	7,259	1
Culture-negative pneumonia	89,794	66	77,211	14
FEMALE	72,761	54	339,590	62
AGE in years: Mean (standard deviation)	68 (19)		40 (17)	
≥ 18–39	12,221	9	310,654	57
≥ 40–49	9,754	7	83,587	15
≥ 50–59	19,062	14	77,044	14
≥ 60–69	23,764	18	42,157	8
≥ 70–79	26,816	20	21,425	4
≥ 80	43,619	32	14,661	3
RACE/ETHNICITY				
White	44,474	48	41,958	27
Black	23,037	25	59,746	39
American Indian	204	0	503	0
Asian	5,122	6	7,420	5
Native Hawaiian	70	0	140	0
Hispanic	19,602	21	43,765	29
YEAR				
2014	25,780	19	132,435	24
2015	24,645	18	128,258	23
2016	23,298	17	131,441	24
2017	21,243	16	126,544	23
2018	24,788	18	14,065	3
2019	15,482	11	16,785	3
SEASON				
Spring	36,152	27	152,371	28
Summer	26,390	20	102,730	19
Fall	27,457	20	117,933	21
Winter	45,237	33	176,494	32
Length of stay in days: Mean ± standard deviation	5.3 ± 7.5		0.1 ± 0.8	

**Table 2:** Distribution of PM_2.5_ concentrations (μg/m^3^) by study site and time period for case periods and control periods

		CASES						CONTROLS					
Site	Time Period	5th %tile	25th %tile	50th %tile	75th %tile	95th %tile	Max	5th %tile	25th %tile	50th %tile	75th %tile	95th %tile	Max

Albany	Overall	1.9	4.3	6.5	9.5	15.6	45.4	1.9	4.3	6.5	9.5	15.6	45.4
	2014–2016	1.5	3.8	6.2	9.6	15.8	45.4	1.5	3.8	6.3	9.6	15.8	45.4
	2017–2019	3.4	5.1	6.8	9.3	14.6	31.5	3.3	5.0	6.7	9.3	14.8	31.5
Bronx	Overall	2.4	4.6	6.9	10.2	16.8	33.1	2.3	4.6	6.8	10.2	16.7	33.1
	2014–2016	2.8	4.8	7.0	10.3	17.1	30.2	2.7	4.8	7.0	10.3	16.8	30.2
	2017–2019	1.7	3.7	6.3	9.6	16.3	33.1	1.7	3.7	6.2	9.6	16.0	33.1
Buffalo	Overall	2.4	5.0	7.0	10.0	15.8	29.3	2.3	4.9	7.0	10.1	15.8	29.3
	2014–2016	1.8	4.4	7.1	10.5	16.3	29.1	1.7	4.4	7.1	10.5	16.3	29.1
	2017–2019	4.2	5.4	6.8	9.1	13.1	29.3	4.1	5.3	6.8	9.0	13.1	29.3
Manhattan	Overall	2.9	5.6	8.3	11.5	17.2	33.5	2.9	5.5	8.2	11.4	17.1	33.5
	2014–2016	3.6	6.2	9.2	12.3	18.5	33.5	3.5	6.1	9.1	12.3	18.4	33.5
	2017–2019	2.2	4.4	6.9	9.5	14.2	27.9	2.1	4.3	6.8	9.5	14.0	27.9
Queens	Overall	2.2	4.1	6.2	9.2	15.7	29.0	2.2	4.0	6.2	9.2	15.7	29.0
	2014–2016	2.7	4.5	6.6	9.7	16.7	29.0	2.7	4.5	6.5	9.7	16.7	29.0
	2017–2019	1.4	3.3	5.2	8.1	14.5	27.2	1.3	3.2	5.1	8.0	14.3	27.2
Rochester	Overall	1.9	3.8	5.9	8.8	14.3	24.8	1.9	3.8	5.9	8.8	14.6	24.8
	2014–2016	1.7	3.9	6.2	9.1	15.5	23.9	1.7	3.9	6.1	9.2	15.5	23.9
	2017–2019	2.2	3.6	5.5	8.1	13.2	24.8	2.2	3.6	5.5	8.1	13.2	24.8

**Table 3:** Excess rate of upper and lower respiratory tract infection hospital admissions and emergency department visits associated with interquartile range increases (IQR) in PM_2.5_ concentration by lag time, outcome, and period

Outcome	Lag days	*N* Before	*N* During	IQR (μg/m^3^)	2014–2016 Excess rate % (95% CI)	2017–2019 Excess rate % (95% CI)	*P* value for interaction (2 df)

HOSPITAL ADMISSIONS							
Influenza	0	7,034	7,746	5.5	3.2 (− 0.5, 7.0)	− 0.3 (− 3.5, 3.0)	0.154
	0–1	7,018	7,605	5.4	4.1 (− 0.2, 8.6)	1.8 (− 2.0, 5.8)	0.432
	0–2	7,001	7,474	4.7	3.8 (− 0.4, 8.3)	3.3 (− 0.7, 7.5)	0.858
	0–3	6,974	7,333	4.4	5.0 (0.5, 9.7)	4.5 (0.2, 9.1)	0.872
	0–4	6,952	7,191	4.0	4.6 (0.3, 9.2)	6.4 (1.9, 11.0)	0.594
	0–5	6,932	7,081	4.1	5.6 (0.7, 10.7)	9.5 (4.3, 14.9)	0.286
	0–6	6,919	6,981	4.1	4.6 (− 0.5, 10.1)	11.9 (6.1, 18.0)	0.070
	7–13	6,769	6,358	4.0	− 8.0 (− 12.5, − 3.3)	12.6 (6.4, 19.1)	0.001
	14–20	6,654	5,829	3.7	− 10.1 (− 14.6, − 5.4)	3.9 (− 1.5, 9.7)	0.001
	21–27	6,554	5,405	3.4	6.8 (1.8, 12.0)	9.7 (4.3, 15.4)	0.444
URI	0	8,268	2,910	5.0	2.8 (− 0.3, 6.0)	1.0 (− 4.3, 6.7)	0.573
	0–1	8,233	2,846	4.6	3.7 (0.2, 7.2)	0.4 (− 5.4, 6.6)	0.344
	0–2	8,207	2,789	4.3	3.3 (− 0.3, 7.0)	− 3.0 (− 9.2, 3.6)	0.089
	0–3	8,185	2,735	3.8	2.9 (− 0.6, 6.6)	− 4.3 (− 10.6, 2.4)	0.051
	0–4	8,150	2,680	3.8	2.8 (− 1.1, 6.8)	− 4.0 (− 11.1, 3.7)	0.111
	0–5	8,120	2,640	3.7	1.3 (− 2.7, 5.4)	− 2.6 (− 10.1, 5.6)	0.385
	0–6	8,090	2,606	3.7	0.7 (− 3.5, 5.1)	− 4.3 (− 12.4, 4.6)	0.302
	7–13	7,907	2,315	3.8	− 0.1 (− 4.4, 4.3)	0.2 (− 9.0, 10.2)	0.951
	14–20	7,756	2,127	3.8	0.6 (− 3.9, 5.3)	3.6 (− 6.1, 14.2)	0.584
	21–27	7,589	1,986	3.7	0.2 (− 4.2, 4.8)	− 2.1 (− 11.7, 8.5)	0.677
Bacterial pneumonia	0	7,991	8,243	4.8	1.3 (− 1.6, 4.3)	− 2.4 (− 5.6, 0.9)	0.080
	0–1	7,955	8,121	4.1	0.9 (− 2.0, 4.0)	− 2.3 (− 5.5, 1.0)	0.134
	0–2	7,926	8,034	3.9	0.6 (− 2.5, 3.8)	− 2.7 (− 6.2, 0.9)	0.153
	0–3	7,905	7,905	3.6	0.2 (− 2.9, 3.4)	− 3.0 (− 6.5, 0.7)	0.178
	0–4	7,880	7,816	3.5	− 0.1 (− 3.4, 3.3)	− 2.2 (− 6.0, 1.8)	0.402
	0–5	7,851	7,722	3.3	− 0.8 (− 4.1, 2.7)	− 1.8 (− 5.7, 2.3)	0.692
	0–6	7,818	7,633	3.2	− 1.7 (− 5.1, 1.8)	− 1.4 (− 5.5, 2.9)	0.894
	7–13	7,630	7,059	3.1	− 1.2 (− 4.5, 2.2)	0.0 (− 4.4, 4.7)	0.658
	14–20	7,440	6,618	3.1	0.8 (− 2.7, 4.4)	1.1 (− 3.6, 6.1)	0.906
	21–27	7,257	6,265	3.1	3.8 (0.0, 7.7)	0.6 (− 4.3, 5.8)	0.309
Culture-negative pneumonia	0	49,318	34,405	5.1	1.3 (− 0.0, 2.6)	1.8 (0.1, 3.5)	0.604
	0–1	49,103	33,898	4.5	1.8 (0.5, 3.2)	2.4 (0.7, 4.2)	0.590
	0–2	48,953	33,399	4.2	2.5 (1.1, 3.9)	3.1 (1.2, 5.0)	0.616
	0–3	48,770	32,899	3.9	2.3 (0.9, 3.8)	3.4 (1.4, 5.4)	0.383
	0–4	48,602	32,439	3.7	2.2 (0.7, 3.7)	3.7 (1.7, 5.9)	0.221
	0–5	48,444	32,015	3.5	1.7 (0.2, 3.3)	3.9 (1.8, 6.1)	0.094
	0–6	48,283	31,614	3.4	1.2 (− 0.4, 2.7)	3.9 (1.6, 6.2)	0.048
	7–13	47,178	28,925	3.4	− 2.8 (− 4.3, − 1.3)	− 1.2 (− 3.6, 1.3)	0.249
	14–20	46,126	26,789	3.4	− 0.0 (− 1.6, 1.6)	− 2.0 (− 4.5, 0.6)	0.189
	21–27	45,048	25,073	3.5	2.3 (0.6, 4.0)	0.0 (− 2.7, 2.8)	0.147
EMERGENCY DEPARTMENT VISITS							
Influenza	0	20,620	7,114	5.5	4.7 (2.6, 7.0)	0.5 (− 3.2, 4.4)	0.058
	0–1	20,532	7,011	4.6	4.8 (2.6, 7.0)	− 1.3 (− 5.1, 2.7)	0.008
	0–2	20,444	6,909	4.6	6.7 (4.2, 9.3)	− 0.2 (− 4.9, 4.7)	0.013
	0–3	20,346	6,802	4.7	7.5 (4.6, 10.4)	2.8 (− 3.1, 9.0)	0.168
	0–4	20,260	6,704	4.4	6.7 (3.7, 9.7)	3.7 (− 2.6, 10.4)	0.411
	0–5	20,163	6,618	4.7	6.8 (3.5, 10.3)	3.7 (− 3.3, 11.3)	0.452
	0–6	20,060	6,524	4.9	6.4 (2.7, 10.2)	4.4 (− 3.3, 12.8)	0.669
	7–13	19,515	6,011	4.8	− 6.3 (− 9.5, − 2.9)	5.7 (− 2.6, 14.6)	0.008
	14–20	19,100	5,575	4.7	− 3.6 (− 7.1, 0.0)	− 2.2 (− 9.6, 5.7)	0.749
	21–27	18,743	5,219	4.5	3.9 (0.2, 7.8)	16.8 (8.3, 26.0)	0.006
URI	0	326,096	95,406	5.3	0.8 (0.3, 1.4)	3.0 (1.9, 4.1)	< 0.001
	0–1	324,788	93,475	4.6	0.2 (− 0.3, 0.7)	1.9 (0.8, 3.1)	0.004
	0–2	323,471	91,696	4.2	0.1 (− 0.5, 0.6)	1.7 (0.5, 3.0)	0.012
	0–3	322,237	90,066	3.9	0.0 (− 0.5, 0.6)	2.1 (0.8, 3.4)	0.003
	0–4	321,013	88,467	3.7	0.0 (− 0.6, 0.6)	2.4 (0.9, 3.8)	0.002
	0–5	319,760	86,986	3.5	0.0 (− 0.6, 0.6)	2.2 (0.7, 3.7)	0.006
	0–6	318,722	85,606	3.4	− 0.3 (− 0.9, 0.3)	1.6 (0.1, 3.2)	0.021
	7–13	311,667	75,995	3.5	− 1.5 (− 2.1, − 0.9)	0.2 (− 1.5, 1.9)	0.061
	14–20	304,708	69,152	3.6	0.4 (− 0.3, 1.1)	1.0 (− 0.7, 2.8)	0.504
	21–27	297,603	63,922	3.6	2.1 (1.4, 2.8)	5.2 (3.3, 7.1)	0.003
Bacterial pneumonia	0	3,353	3,380	5.0	6.7 (1.7, 12.0)	8.6 (3.0, 14.6)	0.612
	0–1	3,342	3,339	4.4	6.6 (1.7, 11.9)	5.7 (− 0.0, 11.7)	0.802
	0–2	3,328	3,307	4.0	7.3 (2.2, 12.6)	2.6 (− 3.2, 8.8)	0.230
	0–3	3,317	3,265	4.0	7.1 (1.5, 13.1)	1.4 (− 5.2, 8.5)	0.196
	0–4	3,309	3,227	3.9	5.4 (− 0.4, 11.5)	2.2 (− 4.9, 9.7)	0.485
	0–5	3,301	3,193	3.9	4.6 (− 1.5, 11.2)	3.1 (− 4.4, 11.3)	0.763
	0–6	3,296	3,158	3.8	3.0 (− 3.5, 9.9)	0.8 (− 7.2, 9.5)	0.679
	7–13	3,245	2,952	3.6	− 3.2 (− 8.9, 2.9)	4.1 (− 4.4, 13.4)	0.164
	14–20	3,200	2,781	3.4	3.6 (− 2.3, 9.9)	1.1 (− 7.1, 10.1)	0.632
	21–27	3,148	2,645	3.1	6.4 (1.0, 12.2)	− 3.1 (− 10.6, 4.9)	0.045
Culture-negative pneumonia	0	35,066	37,006	4.9	0.2 (− 1.2, 1.7)	2.2 (0.7, 3.8)	0.053
	0–1	34,927	36,521	4.3	0.7 (− 0.8, 2.2)	3.0 (1.4, 4.6)	0.031
	0–2	34,798	36,032	3.9	0.6 (− 0.9, 2.1)	4.3 (2.5, 6.0)	0.001
	0–3	34,664	35,516	3.7	0.8 (− 0.7, 2.4)	5.4 (3.5, 7.2)	< 0.001
	0–4	34,554	35,054	3.5	0.8 (− 0.7, 2.4)	6.5 (4.5, 8.4)	< 0.001
	0–5	34,433	34,662	3.4	0.9 (− 0.7, 2.6)	6.9 (4.9, 8.9)	< 0.001
	0–6	34,309	34,278	3.2	0.8 (− 0.9, 2.5)	7.8 (5.7, 9.9)	< 0.001
	7–13	33,489	31,749	3.3	− 0.5 (− 2.2, 1.2)	− 0.2 (− 2.4, 2.1)	0.826
	14–20	32,735	29,645	3.3	− 1.8 (− 3.5, − 0.0)	− 1.0 (− 3.4, 1.4)	0.611
	21–27	31,968	27,946	3.2	2.1 (0.3, 4.0)	− 1.1 (− 3.5, 1.4)	0.031

**Table 4:** Excess rate of influenza hospital admissions and emergency department visits associated with interquartile range (IQR) increases in PM_2.5_ concentration by lag time, outcome, period, and sex

Outcome	Lag days	*N* Before	*N* During	IQR (μg/m^3^)	2014–2016 Excess rate % (95% CI)	2017–2019 Excess rate % (95% CI)	*P* value for interaction (2 df)

HOSPITAL ADMISSIONS							
Influenza—male	0	2,999	3,143	5.6	1.2 (− 4.4, 7.1)	1.8 (− 3.4, 7.3)	0.877
	0–1	2,990	3,094	5.5	0.6 (− 5.8, 7.5)	5.0 (− 1.2, 11.6)	0.340
	0–2	2,984	3,044	4.6	0.1 (− 6.0, 6.5)	4.8 (− 1.3, 11.2)	0.286
	0–3	2,976	2,981	4.4	− 0.1 (− 6.6, 6.8)	6.9 (0.0, 14.2)	0.151
	0–4	2,971	2,922	4.3	− 1.6 (− 8.3, 5.6)	7.5 (− 0.0, 15.6)	0.085
	0 – 5	2,965	2,884	4.0	− 0.6 (− 7.4, 6.6)	8.6 (0.9, 16.8)	0.085
	0–6	2,959	2,836	4.3	− 0.7 (− 8.4, 7.7)	10.4 (1.3, 20.3)	0.077
	7–13	2,888	2,585	4.0	− 8.2 (− 15.0, − 0.9)	6.9 (− 2.2, 16.8)	0.012
	14–20	2,835	2,354	3.9	− 8.1 (− 15.1, − 0.5)	1.6 (− 6.8, 10.7)	0.097
	21–27	2,790	2,175	3.4	4.4 (− 3.0, 12.4)	13.9 (5.4, 23.0)	0.112
Influenza—female	0	8,268	2,910	5.0	4.7 (− 0.1, 9.8)	− 1.7 (− 5.8, 2.5)	0.045
	0–1	8,233	2,846	4.6	6.8 (1.1, 12.9)	− 0.3 (− 5.1, 4.8)	0.065
	0–2	8,207	2,789	4.3	7.0 (1.0, 13.4)	2.3 (− 3.0, 7.9)	0.254
	0–3	8,185	2,735	3.8	9.2 (3.0, 15.9)	3.0 (− 2.7, 9.0)	0.153
	0–4	8,150	2,680	3.8	9.5 (3.4, 15.9)	6.1 (0.3, 12.4)	0.449
	0–5	8,120	2,640	3.7	10.6 (3.8, 17.8)	10.2 (3.4, 17.4)	0.934
	0–6	8,090	2,606	3.7	8.8 (1.7, 16.4)	13.5 (5.8, 21.6)	0.393
	7–13	7,907	2,315	3.8	− 7.4 (− 13.1, − 1.4)	15.7 (8.0, 24.0)	< 0.001
	14–20	7,756	2,127	3.8	− 11.9 (− 17.8, − 5.7)	5.8 (− 1.4, 13.6)	< 0.001
	21–27	7,589	1,986	3.7	8.7 (2.0, 15.8)	6.9 (− 0.0, 14.3)	0.731
EMERGENCY DEPARTMENT VISITS							
Influenz—male	0	8,344	2,885	5.4	4.6 (1.2, 8.1)	0.4 (− 5.4, 6.5)	0.224
	0–1	8,315	2,845	4.6	4.5 (1.0, 8.0)	− 4.3 (− 10.1, 1.9)	0.013
	0–2	8,288	2,806	4.5	6.6 (2.7, 10.6)	− 5.2 (− 12.1, 2.2)	0.005
	0–3	8,254	2,747	4.5	8.1 (3.7, 12.6)	− 2.0 (− 10.5, 7.3)	0.049
	0–4	8,212	2,708	4.3	8.6 (4.1, 13.3)	− 0.0 (− 9.3, 10.1)	0.121
	0–5	8,176	2,683	4.6	9.3 (4.0, 14.8)	− 0.6 (− 11.1, 11.0)	0.121
	0–6	8,133	2,642	4.4	8.1 (2.8, 13.8)	− 3.3 (− 13.6, 8.2)	0.072
	7–13	7,927	2,433	4.6	− 5.0 (− 9.9, 0.2)	1.9 (− 9.9, 15.2)	0.310
	14–20	7,755	2,266	4.7	− 6.3 (− 11.6, − 0.7)	4.0 (− 7.9, 17.4)	0.130
	21–27	7,610	2,123	4.4	5.9 (0.1, 12.1)	18.8 (5.5, 33.8)	0.086
Influenza—female	0	12,275	4,229	5.5	4.9 (2.0, 7.8)	0.6 (− 4.3, 5.7)	0.140
	0–1	12,216	4,166	4.7	5.1 (2.2, 8.1)	0.8 (− 4.3, 6.2)	0.159
	0–2	12,155	4,103	4.7	6.9 (3.5, 10.3)	3.4 (− 2.9, 10.1)	0.353
	0–3	12,091	4,055	4.8	6.9 (3.2, 10.9)	6.1 (− 1.7, 14.6)	0.852
	0–4	12,047	3,996	4.5	5.3 (1.5, 9.3)	6.4 (− 2.0, 15.4)	0.827
	0–5	11,986	3,935	4.9	5.4 (0.9, 10.1)	7.0 (− 2.7, 17.6)	0.773
	0–6	11,926	3,882	5.0	4.8 (− 0.1, 9.9)	10.2 (− 0.4, 22.0)	0.371
	7–13	11,587	3,578	4.9	− 7.0 (− 11.2, − 2.6)	8.2 (− 2.6, 20.3)	0.010
	14–20	11,344	3,309	4.8	− 1.7 (− 6.4, 3.2)	− 6.3 (− 15.4, 3.8)	0.408
	21–27	11,132	3,096	4.7	2.6 (− 2.4, 7.8)	15.9 (4.7, 28.2)	0.034

## Data Availability

The datasets generated during and/or analyzed during the current study are not publicly available due to the training and registration requirements of the Statewide Planning and Research Cooperative System (SPARCS)database. Queries can be addressed via https://www.health.ny.gov/statistics/sparcs/
