# Supplementary material for: Change in rate of healthcare encounters for respiratory infection from air pollution exposure after improved vehicle emissions standards in New York State
Source: Air Qual Atmos Health. Author manuscript; Available in PMC 2026 Feb 21. (PMC12922777; doi:10.1007/s11869-024-01505-6)
Supplement: Supplement [file NIHMS2131625-supplement-Supplement.pdf]

Supplement: Change in rate of healthcare encounters for respiratory infection from air pollution exposure after improved vehicle emissions standards in New York State

Daniel P. Croft MD, MPH,[1] Mark J Utell, MD,[1,2] Han Liu,[3] Shao Lin MD, PhD,[4]

Philip K. Hopke, PhD,[5,6] Sally W. Thurston, PhD,[7] Yunle Chen, PhD,[5]

David Q Rich, ScD [1,2,5]

1. Department of Medicine, University of Rochester Medical Center, Rochester, New York.
2. Department of Environmental Medicine, University of Rochester Medical Center, Rochester, New York
3. Department of Sociology, University at Albany, the State University of New York, Albany, New York
4. Department of Environmental Health Sciences. University at Albany, the State University of New York, Albany, New York
5. Department of Public Health Sciences, University of Rochester Medical Center, Rochester, New York
6. Institute for a Sustainable Environment, and Center for Air Resources Engineering and Science, Clarkson University, Potsdam, New York
7. Department of Biostatistics and Computational Biology, University of Rochester Medical Center, Rochester, New York

Corresponding author:
Daniel P Croft
Assistant Professor
E-mail: daniel_croft@urmc.rochester.edu.
Phone: 585 275 4161. Fax: 585 271 1171.
Strong Memorial Hospital
601 Elmwood Avenue, Box 692
Rochester, NY 14642-8692

Table S1. Excess rate of upper respiratory tract infection hospital admissions and emergency department visits associated with interquartile range increases in fine particulate matter air pollution (2.5 µm or less in diameter concentration), by lag time, outcome, period and sex.

Outcome	Lag Days	N Before	N During	IQR (µg/m^3)	2014-2016 Excess rate % (95% CI)	2017-2019 Excess rate % (95% CI)	p-value for interaction (2 df)
**HOSPITAL ADMISSIONS (Adult)**							
URI-male	0	3,278	1,239	5.0	4.0 (-0.8, 9.0)	6.0 (-2.4, 15.2)	0.676
	0-1	3,264	1,216	4.6	5.6 (0.3, 11.2)	2.8 (-6.2, 12.6)	0.592
	0-2	3,250	1,191	4.1	5.2 (-0.1, 10.9)	-4.0 (-12.9, 5.8)	0.087
	0-3	3,242	1,168	3.7	5.5 (0.1, 11.2)	-5.4 (-14.4, 4.6)	0.050
	0-4	3,228	1,142	3.8	6.7 (0.6, 13.2)	-7.7 (-17.8, 3.8)	0.025
	0-5	3,211	1,125	3.7	5.0 (-1.3, 11.7)	-9.5 (-20.0, 2.5)	0.030
	0-6	3,194	1,110	3.6	5.4 (-1.3, 12.6)	-13.9 (-24.8, -1.4)	0.006
	7-13	3,112	996	3.7	0.4 (-6.1, 7.3)	8.2 (-5.9, 24.3)	0.333
	14-20	3,049	922	3.7	0.2 (-6.5, 7.4)	2.2 (-11.2, 17.7)	0.797
	21-27	2,994	854	3.5	1.7 (-4.7, 8.7)	-10.7 (-22.9, 3.4)	0.102
URI-female	0	4,990	1,671	5.1	2.0 (-2.1, 6.3)	-2.5 (-9.4, 4.9)	0.273
	0-1	4,969	1,630	4.7	2.2 (-2.1, 6.8)	-1.2 (-8.7, 6.9)	0.441

	0-2	4,957	1,598	4.4	1.7 (-2.9, 6.7)	-2.0 (-10.4, 7.2)	0.450
	0-3	4,943	1,567	3.8	1.0 (-3.5, 5.8)	-3.4 (-11.7, 5.7)	0.364
	0-4	4,922	1,538	3.8	0.1 (-4.7, 5.2)	-1.0 (-10.5, 9.5)	0.841
	0-5	4,909	1,515	3.8	-1.2 (-6.2, 4.1)	3.1 (-7.4, 14.8)	0.479
	0-6	4,896	1,496	3.8	-2.4 (-7.8, 3.2)	4.0 (-7.5, 16.9)	0.327
	7-13	4,795	1,319	3.9	-0.5 (-6.1, 5.4)	-6.0 (-17.5, 7.2)	0.429
	14-20	4,707	1,205	3.9	0.9 (-5.1, 7.3)	5.5 (-8.0, 20.9)	0.553
	21-27	4,595	1,132	3.7	-0.9 (-6.6, 5.1)	6.2 (-7.7, 22.3)	0.362
**EMERGENCY DEPARTMENT VISITS (Adults)**							
URI-male	0	118,672	34,747	5.3	0.4 (-0.5, 1.2)	4.2 (2.5, 6.0)	<0.001
	0-1	118,199	34,064	4.6	-0.0 (-0.9, 0.8)	2.5 (0.7, 4.4)	0.011
	0-2	117,741	33,427	4.2	-0.0 (-0.9, 0.9)	1.7 (-0.3, 3.7)	0.112
	0-3	117,285	32,836	3.8	0.0 (-0.9, 0.9)	1.9 (-0.3, 4.1)	0.109
	0-4	116,837	32,265	3.7	0.0 (-0.9, 1.0)	2.2 (-0.1, 4.6)	0.078
	0-5	116,400	31,749	3.5	-0.2 (-1.1, 0.8)	1.8 (-0.6, 4.3)	0.122
	0-6	116,037	31,236	3.4	-0.4 (-1.4, 0.6)	0.6 (-1.9, 3.2)	0.445
	7-13	113,498	27,696	3.5	-0.9 (-1.9, 0.1)	0.4 (-2.4, 3.2)	0.368
	14-20	110,995	25,230	3.7	0.6 (-0.5, 1.7)	1.3 (-1.7, 4.3)	0.656

	21-27	108,431	23,327	3.6	1.7 (0.6, 2.9)	5.3 (2.2, 8.5)	0.030
URI-female	0	207,418	60,658	5.2	1.1 (0.4, 1.7)	2.3 (0.9, 3.6)	0.104
	0-1	206,584	59,410	4.6	0.3 (-0.3, 1.0)	1.6 (0.2, 3.0)	0.093
	0-2	205,725	58,268	4.2	0.1 (-0.5, 0.8)	1.7 (0.2, 3.3)	0.053
	0-3	204,947	57,229	3.9	0.0 (-0.7, 0.7)	2.2 (0.5, 3.9)	0.015
	0-4	204,171	56,201	3.7	0.0 (-0.7, 0.7)	2.5 (0.6, 4.3)	0.011
	0-5	203,355	55,236	3.5	0.1 (-0.6, 0.9)	2.4 (0.5, 4.3)	0.023
	0-6	202,680	54,369	3.4	-0.3 (-1.0, 0.5)	2.1 (0.2, 4.1)	0.021
	7-13	198,164	48,298	3.5	-1.8 (-2.6, -1.1)	0.0 (-2.1, 2.2)	0.096
	14-20	193,708	43,921	3.6	0.3 (-0.5, 1.1)	0.9 (-1.3, 3.1)	0.616
	21-27	189,167	40,594	3.6	2.4 (1.5, 3.2)	5.1 (2.7, 7.5)	0.032

Table S2. Excess rate of bacterial pneumonia hospital admissions and emergency department visits associated with interquartile range increases in fine particulate matter air pollution (2.5 µm or less in diameter concentration), by lag time, outcome, period and sex.

Outcome	Lag Days	N Before	N During	IQR ($\mu g/m^3$)	2014-2016 Excess rate % (95% CI)	2017-2019 Excess rate % (95% CI)	p-value for interaction (2 df)

**HOSPITAL ADMISSIONS (Adult)**							
Bacterial pneumonia-male	0	3,867	3,973	4.8	-0.0 (-4.2, 4.3)	-3.4 (-7.9, 1.3)	0.264
	0-1	3,849	3,917	4.1	0.6 (-3.7, 5.0)	-2.5 (-7.1, 2.3)	0.321
	0-2	3,838	3,874	4.0	0.1 (-4.4, 4.9)	-2.6 (-7.6, 2.7)	0.429
	0-3	3,828	3,812	3.6	-0.9 (-5.4, 3.8)	-2.8 (-7.9, 2.6)	0.581
	0-4	3,817	3,772	3.5	-1.5 (-6.1, 3.5)	-2.9 (-8.3, 2.8)	0.683
	0-5	3,803	3,731	3.3	-2.3 (-6.9, 2.6)	-2.4 (-7.9, 3.5)	0.975
	0-6	3,787	3,688	3.1	-3.1 (-7.9, 1.8)	-2.4 (-8.2, 3.7)	0.852
	7-13	3,704	3,409	3.0	-1.0 (-5.6, 3.9)	-3.0 (-9.1, 3.4)	0.598
	14-20	3,615	3,184	3.1	2.2 (-2.9, 7.6)	6.4 (-0.7, 14.1)	0.335
	21-27	3,523	3,015	3.2	9.8 (4.0, 15.8)	1.3 (-5.9, 9.0)	0.071
Bacterial pneumonia - female	0	4,124	4,270	4.8	2.6 (-1.5, 6.8)	-1.5 (-6.0, 3.2)	0.174
	0-1	4,106	4,204	4.1	1.3 (-2.8, 5.6)	-2.1 (-6.6, 2.6)	0.257
	0-2	4,088	4,160	3.8	1.0 (-3.2, 5.4)	-2.8 (-7.5, 2.1)	0.220
	0-3	4,077	4,093	3.6	1.3 (-3.1, 5.9)	-3.2 (-8.1, 2.1)	0.185
	0-4	4,063	4,044	3.5	1.2 (-3.3, 6.0)	-1.5 (-6.8, 4.2)	0.444
	0-5	4,048	3,991	3.3	0.7 (-3.9, 5.5)	-1.2 (-6.7, 4.6)	0.604
	0-6	4,031	3,945	3.2	-0.3 (-5.1, 4.6)	-0.2 (-6.0, 5.9)	0.978

	7-13	3,926	3,650	3.1	-1.5 (-6.1, 3.4)	3.2 (-3.3, 10.0)	0.247
	14-20	3,825	3,434	3.1	-0.5 (-5.2, 4.5)	-3.6 (-9.9, 3.0)	0.423
	21-27	3,734	3,250	3.1	-1.4 (-6.3, 3.7)	-0.0 (-6.7, 7.1)	0.741
**EMERGENCY DEPARTMENT VISITS (Adults)**							
Bacterial pneumonia - male	0	1,587	1,604	4.7	3.3 (-3.2, 10.2)	5.7 (-1.6, 13.6)	0.614
	0-1	1,581	1,580	4.6	3.5 (-3.8, 11.2)	3.6 (-4.7, 12.6)	0.980
	0-2	1,571	1,563	4.1	2.7 (-4.6, 10.6)	-0.3 (-8.6, 8.8)	0.595
	0-3	1,562	1,545	4.1	1.5 (-6.4, 10.2)	-4.3 (-13.4, 5.7)	0.343
	0-4	1,556	1,523	4.1	-0.4 (-8.8, 8.9)	-4.4 (-14.4, 6.7)	0.546
	0-5	1,555	1,507	3.9	-1.4 (-10.0, 8.0)	-2.7 (-13.1, 9.0)	0.862
	0-6	1,552	1,492	4.0	-2.7 (-12.0, 7.4)	-3.4 (-14.7, 9.3)	0.927
	7-13	1,519	1,394	3.8	-3.0 (-11.6, 6.5)	6.4 (-6.3, 20.9)	0.233
	14-20	1,493	1,309	3.5	7.8 (-1.3, 17.7)	-2.6 (-14.2, 10.5)	0.176
	21-27	1,476	1,250	2.8	8.8 (1.6, 16.4)	-8.3 (-17.3, 1.7)	0.005
Bacterial pneumonia - female	0	1,766	1,775	5.2	10.1 (2.8, 17.8)	11.5 (3.2, 20.4)	0.798
	0-1	1,761	1,758	4.3	9.6 (2.7, 16.9)	7.7 (-0.2, 16.2)	0.721
	0-2	1,757	1,743	3.7	10.7 (4.0, 17.8)	4.9 (-2.7, 13.2)	0.262
	0-3	1,755	1,719	3.7	11.1 (3.8, 18.9)	6.3 (-2.4, 15.7)	0.405

0-4	1,753	1,703	3.6	9.9 (2.2, 18.2)	7.8 (-1.7, 18.2)	0.733
0-5	1,746	1,685	3.7	9.9 (1.4, 19.0)	8.5 (-2.1, 20.2)	0.844
0-6	1,744	1,665	3.7	7.8 (-1.0, 17.4)	4.6 (-6.4, 16.9)	0.664
7-13	1,726	1,557	3.6	-3.2 (-10.9, 5.1)	2.1 (-9.2, 14.8)	0.452
14-20	1,707	1,471	3.5	0.2 (-7.8, 8.9)	4.3 (-7.5, 17.5)	0.579
21-27	1,672	1,394	3.4	3.7 (-4.2, 12.2)	2.8 (-8.7, 15.7)	0.898

Table S3. Excess rate of culture negative pneumonia hospital admissions and emergency department visits associated with interquartile range increases in fine particulate matter air pollution (2.5 µm or less in diameter concentration), by lag time, outcome, period and sex.

Outcome	Lag Days	N Before	N During	IQR ($\mu g/m^3$)	2014-2016 Excess rate % (95% CI)	2017-2019 Excess rate % (95% CI)	p-value for interaction (2 df)
**HOSPITAL ADMISSIONS (Adult)**							
Culture negative pneumonia-male	0	23,352	16,325	5.0	1.9 (0.0, 3.8)	1.7 (-0.6, 4.2)	0.932
	0-1	23,240	16,079	4.5	2.4 (0.4, 4.4)	2.5 (-0.0, 5.0)	0.950
	0-2	23,163	15,839	4.1	3.1 (1.0, 5.2)	3.4 (0.7, 6.2)	0.843
	0-3	23,081	15,573	3.8	3.0 (1.0, 5.1)	3.6 (0.8, 6.5)	0.742
	0-4	22,998	15,376	3.6	2.9 (0.8, 5.0)	4.0 (1.1, 7.1)	0.520
	0-5	22,935	15,170	3.4	2.1 (-0.0, 4.3)	3.9 (0.8, 7.1)	0.340
	0-6	22,867	14,998	3.3	1.6 (-0.5, 3.9)	3.9 (0.7, 7.1)	0.251
	7-13	22,350	13,689	3.3	-2.6 (-4.8, -0.5)	1.4 (-2.1, 5.0)	0.048
	14-20	21,826	12,654	3.4	-1.8 (-4.1, 0.5)	-5.9 (-9.4, -2.3)	0.054
	21-27	21,302	11,807	3.5	2.3 (-0.2, 4.8)	0.5 (-3.4, 4.6)	0.442
Culture negative pneumonia - female	0	25,966	18,080	5.2	0.7 (-1.1, 2.5)	1.8 (-0.5, 4.2)	0.428
	0-1	25,863	17,819	4.4	1.4 (-0.4, 3.2)	2.4 (0.0, 4.8)	0.491
	0-2	25,790	17,560	4.2	2.0 (0.0, 3.9)	2.7 (0.2, 5.4)	0.622

	0-3	25,689	17,326	3.9	1.7 (-0.3, 3.7)	3.2 (0.5, 6.0)	0.371
	0-4	25,604	17,063	3.7	1.6 (-0.5, 3.6)	3.5 (0.6, 6.4)	0.279
	0-5	25,509	16,845	3.5	1.4 (-0.7, 3.5)	3.9 (0.9, 7.0)	0.159
	0-6	25,416	16,616	3.4	0.7 (-1.4, 2.9)	3.8 (0.7, 7.0)	0.100
	7-13	24,828	15,236	3.4	-3.0 (-5.1, -0.9)	-3.6 (-6.9, -0.2)	0.770
	14-20	24,300	14,135	3.5	1.7 (-0.6, 4.0)	1.7 (-2.0, 5.5)	0.993
	21-27	23,746	13,266	3.4	2.3 (0.1, 4.7)	-0.4 (-4.0, 3.4)	0.205
**EMERGENCY DEPARTMENT VISITS (Adults)**							
Culture negative pneumonia - male	0	16,453	17,101	4.8	1.0 (-1.1, 3.1)	1.8 (-0.5, 4.1)	0.599
	0-1	16,396	16,865	4.2	1.3 (-0.8, 3.5)	1.7 (-0.6, 4.1)	0.785
	0-2	16,334	16,639	3.9	0.7 (-1.4, 3.0)	2.5 (0.1, 5.0)	0.270
	0-3	16,281	16,401	3.7	1.2 (-1.1, 3.5)	3.6 (1.0, 6.3)	0.158
	0-4	16,233	16,190	3.5	0.9 (-1.4, 3.3)	5.1 (2.3, 8.0)	0.022
	0-5	16,172	16,009	3.4	0.7 (-1.7, 3.1)	6.4 (3.5, 9.4)	0.002
	0-6	16,115	15,837	3.2	0.4 (-2.1, 2.9)	7.8 (4.7, 10.9)	<0.001
	7-13	15,731	14,662	3.3	-0.6 (-3.0, 1.9)	-0.9 (-4.1, 2.5)	0.894
	14-20	15,385	13,698	3.3	-1.4 (-3.9, 1.2)	-3.6 (-7.0, -0.1)	0.294
	21-27	15,035	12,890	3.3	3.1 (0.5, 5.9)	-1.3 (-4.8, 2.4)	0.047

Culture negative pneumonia - female	0	18,613	19,902	4.9	-0.4 (-2.4, 1.6)	2.7 (0.5, 4.9)	0.031
	0-1	18,531	19,653	4.4	0.1 (-1.9, 2.1)	4.1 (1.8, 6.4)	0.007
	0-2	18,464	19,390	3.9	0.5 (-1.6, 2.6)	5.8 (3.4, 8.2)	0.001
	0-3	18,383	19,112	3.7	0.4 (-1.7, 2.6)	6.8 (4.3, 9.4)	0.001
	0-4	18,321	18,861	3.5	0.8 (-1.4, 3.0)	7.8 (5.1, 10.5)	<0.001
	0-5	18,261	18,650	3.4	1.1 (-1.1, 3.4)	7.3 (4.6, 10.2)	<0.001
	0-6	18,194	18,438	3.2	1.1 (-1.2, 3.5)	7.8 (5.0, 10.8)	<0.001
	7-13	17,758	17,084	3.3	-0.4 (-2.7, 1.9)	0.4 (-2.7, 3.5)	0.685
	14-20	17,350	15,944	3.3	-2.1 (-4.4, 0.3)	1.1 (-2.1, 4.5)	0.103
	21-27	16,933	15,053	3.2	1.3 (-1.1, 3.8)	-0.9 (-4.1, 2.4)	0.278
